# Rap1 Guanosine Triphosphate Hydrolase (GTPase) Regulates Shear Stress-Mediated Adhesion of Mesenchymal Stromal Cells

**DOI:** 10.3390/biology14010096

**Published:** 2025-01-18

**Authors:** Melanie Giesen, Erika Fleck, Jürgen Scheele, Tanja Nicole Hartmann, Reinhard Henschler

**Affiliations:** 1 Institute for Transfusion Medicine and Immune Hematology, German Red Cross Blood Donor Service, Clinics of the Goethe University Frankfurt (Main), 60528 Frankfurt am Main, Germany; melanie.giesen@biontech.de (M.G.); e.fleck@blutspende.de (E.F.); 2 Department of Medicine I, Medical Center University of Freiburg and Faculty of Medicine, University of Freiburg, 79106 Freiburg, Germany; juergen.scheele@pharmakol.uni-freiburg.de (J.S.); tanja.hartmann@uniklinik-freiburg.de (T.N.H.); 3 Institute for Transfusion Medicine, Medical Faculty, Leipzig University, 04103 Leipzig, Germany

**Keywords:** mesenchymal stromal cells, Rap1, adhesion, integrins, chemokine receptors

## Abstract

Intravenously transplanted mesenchymal stromal cells (MSCs) have been shown to interact with endothelial cells and to migrate to tissues. However, intracellular signals regulating MSC migration are still incompletely understood. Here, we analyzed the role of Rap1 GTPase in the migration of human bone marrow-derived MSCs in vitro and in short-term homing in mice in vivo. MSCs expressed both Rap1A and Rap1B mRNAs, which were downregulated after treatment with siRNA against Rap1A and/or B. In a flow chamber model with pre-established human umbilical vein endothelial cells (HUVECs), Rap1A/B downregulated MSCs interacted for longer distances before arrest, indicating adhesion defects. CXCL12-induced adhesion of MSCs on immobilized Vascular Cell Adhesion Molecule (VCAM)-1 was also decreased after the downregulation of Rap1A, Rap1B, or both, as was CXCL12-induced transwell migration. In a competitive murine short-term homing model with i.v. co-injection of Rap1A+B siRNA-treated and control MSCs that were labeled with PKH 26 and PKH 67 fluorescent dyes, the Rap1A+B siRNA-treated MSCs were detected at increased frequencies in blood, liver, and spleen compared to control MSCs. Thus, Rap1 GTPase modulates the adhesion and migration of MSCs in vitro and may increase the bio-availability of i.v.-transplanted MSCs in tissues in a murine model.

## Simple Summary

Rap1 has been implicated in the migration regulation of mesenchymal stem cells (MSCs). We found both Rap1A and Rap1B mRNAs expressed in human MSCs and suppressed their expression using siRNAs against both isoforms. Transwell migration, shear stress-dependent interaction, and adhesion to endothelial cells were all reduced in MSCs after the downregulation of Rap1A and/or Rap1B. In contrast, in a short-term homing model in immune-suppressed mice, MSCs treated with Rap1A+B siRNA showed increased accumulation in blood, liver, and spleen. Our data suggest that the modulation of Rap1A and/or B interferes with the adhesion, migration, and in vivo circulation behavior of human MSCs.

## 1. Introduction

Intravenously applied MSCs are used as a source for cellular therapies for an increasing number of indications and are considered safe [1]. Some, but still limited, knowledge exists on the molecular mechanisms underlying the migration and engraftment of these cells. In animal models, transfused MSCs have been observed to interact with platelets, neutrophils, and endothelial cells and to migrate into tissues [2,3,4,5]. Endothelial P-selectin and galectins 1 and 3 on MSCs have been implied in regulating MSC migration [6,7], whereas CD44 was found to contribute to MSC homing only if a glycan modification of the hematopoietic cell E-selectin/L-selectin ligand epitope is enzymatically induced [8]. Integrin-mediated activation and migration in MSCs have been shown to involve the stimulation of chemokine receptors [9,10]. Particularly, integrin α4β1 expressed on MSCs has been described to mediate the interaction of transplanted MSCs with endothelial cells and their intra-tissue environment [11,12,13].

Rho family GTPases have been shown to play a role in the MSCs of Rho family GTPases in MSC migration [14], actin/collagen expression and MSC differentiation [15,16], and MSC fate decisions [17]. A role of Rap1 signaling has been suggested in MSC proliferation and migration, as well as cardiac repair [18,19,20].

It is currently unclear to which degree intravenously transplanted MSCs transmigrate and successfully extravasate into tissues [5,21]. We and others have shown that the migration of MSCs is an active process that involves signaling by Rho GTPases [6,22]. Rap1 is a RAS family small GTPase that has been shown to be involved in the extravasation and transmigration of leukocytes [23]. Here, we aimed to investigate whether the modulation of Rap1 levels in MSCs may affect the adhesion and migration of MSCs under shear stress in vitro and in a short-term homing model in mice.

## 2. Materials and Methods

### 2.1. Antibodies, Reagents, and Cells

MSCs were obtained from bone marrow harvests of patients undergoing hip replacement surgery or from voluntary donors after informed consent and under a procedure approved by the local ethics committee according to the Declaration of Helsinki (vote 102/03). In brief, MSCs were flushed from the filters of a 3-way collection filter system (Fenwal/Fresenius, Bad Homburg, Germany) and subsequently propagated in low-glucose Dulbecco’s Modified Eagle’s Medium (DMEM, PAA/GE Healthcare, Cölbe, Germany) containing 20% fetal bovine serum (Lonza, Basel, Switzerland). MSCs were used for experiments when they reached 80% confluence at passages between 5 and 9. MSCs were tested negative for CD45 (antibody clone H130, BD Bioscience, Heidelberg, Germany) and CD34 (AC136, mouse Ig 2a, Miltenyi Biotec, Bergisch Gladbach, Germany) and positive for CD105 (MCA 1557F, Serotec, Oxford, UK) and CD73 (clone AD2, BD Bioscience) by flow cytometry. Isolated MSCs displayed adipogenic, chondrogenic, and osteogenic differentiation potential using NH AdipoDiff Medium, NH ChondroDiff Medium, and NH OsteoDiff Medium (Miltenyi Biotec, Bergisch Gladbach, Germany). Human umbilical vein endothelial cells (HUVECs; Cambrex, Brussels, Belgium) and peripheral blood mononuclear cells (PBMCs; obtained from normal blood donors after informed consent) were isolated and used as described previously [22].

### 2.2. Cell Transfection and siRNA-Mediated Rap1 Regulation

MSCs were transiently transfected with siRNA using the DharmaFECT Transfection Reagent (Thermo Scientific, Waltham, MA, USA) according to the manufacturer’s instructions. siRNAs (Qiagen, Hilden, Germany) used Rap1A target sequence 5′-CAGGGCCAGAATTTAGCAAGA-3′ (Hs_RAP1A_6), and Rap1B target sequence 5′-GACGAGTACTGTGGATGTGAA-3′ (Hs_RAP1B_6). A non-related, scrambled siRNA (target sequence was 5′-ACG UGA CAC GUU CGG AGA A-3′) was used as control. siRNA-mediated silencing of MSCs was performed in 6-well culture plates (Greiner, Frickenhausen, Germany). MSCs were seeded at 1.5 × 10^5^ cells per well in 2 mL DMEM (Invitrogen, Karlsruhe, Germany) containing 20% FBS and antibiotics. For lipofection, 37.5 ng of Rap 1A and/or Rap1B siRNAs were pre-diluted in 100 µL low-glucose DMEM without serum. Twelve microliters of HiPerFect Transfection Reagent was added to the diluted siRNA and mixed by vortexing. The samples were incubated for 5–10 min at room temperature to allow the formation of the transfection complex and added to the MSCs. After 24 h at 37 °C/5% CO_2_, the culture medium was replaced with fresh low-glucose DMEM/20% FBS. Quantitative real-time PCR was performed using cDNA synthesis reverse transcription kit Omniscript RT (Qiagen) and subsequent qRT-PCR against Rap1A and Rap1B according to the manufacturer’s instructions using primers for Rap1a or Rap1b (Applied Biosystems, Waltham, MA, USA). Expression of Rap1A and Rap1B was found to be maximally suppressed 48 h after siRNA transfection using Western blot analysis. Cells at this time point were used for experiments.

### 2.3. Flow Cytometry and Immunohistochemistry

For analysis of adhesion receptor expression, single-cell suspensions of MSCs were prepared in PBS supplemented with 2% FCS after trypsinization. For cell membrane staining, 1 × 10^6^ cells were directly incubated at 4 °C for 30 min with directly conjugated mAbs against integrin α4 (9EG7) and integrinβ1 (MAR4; both from BD Bioscience). For analysis of chemokine receptor expression, MSCs were fixed in 2% paraformaldehyde for 10 min at room temperature and permeabilized for 30 min in 0.4% saponin (Sigma, Munich, Germany) in PBS, washed in PBS, and subsequently stained with antibodies CCR6 (clone 11A9), CCR7 (clone 3D12), and CXCR4 (clone 12G5, all from BD Pharmingen). The samples were analyzed on a FACS Calibur (Becton Dickinson, Heidelberg, Germany) using CellQuest**™** (Becton Dickinson) and FCS Express (https://denovosoftware.com/; De Novo, Los Angeles, CA, USA) software and live cell gates as previously published [24].

### 2.4. Analysis of MSC Adhesion Under Shear Stress

Adhesion assays of MSCs were performed on parallel-plate flow chambers (Ibitreat VI µ type, Ibidi GmbH, Munich, Germany). Recombinant human (rh) VCAM-1-Fc fusion protein (R&D Systems, Wiesbaden, Germany) was pre-coated at 10 µg/mL 1–2 h before the assays in PBS/1% BSA. For other experiments, chambers were pre-seeded with 1 × 10^6^ HUVECs two days before the experiments. HUVECs were pre-stimulated by the addition of rh TNF-α (tebu-bio, Offenbach, Germany) at a final concentration of 10 ng/mL overnight. Prior to the start of the assays, flow chambers were carefully washed using PBS/1% BSA for 30 min. In some experiments, function-blocking antibody anti-VCAM-1 (clone 1G11, BD Bioscience) or anti-VLA-4 (HP2/1, BD Bioscience) was added at 0.2 mg/mL either to pre-coated slides (anti-VCAM-1) or to MSCs (anti-VLA-4) 30 min before the start of the assay. Rh CXCL12 (R&D Systems) was added at 1 µg/mL in PBS/1% BSA to the flow chambers 10 min prior to the assay. MSCs were trypsinized and kept in suspension at 10^6^ /mL at 37 °C in Hank’s Balanced Salt Solution (HBSS; Invitrogen, Karlsruhe, Germany) supplemented with 0.05% BSA for 30 min prior to the experiments. To study the interaction of flowing cells with endothelial cells or immobilized rhVCAM-1, 10^5^ MSCs in 1 mL HBSS/0.05%BSA were flushed through flow chambers at calculated shear stresses of 0.2–5 dyn/cm^2^. Video recordings were performed using a CCD camera mounted on an inverted-stage microscope (Axiovert 135, Zeiss, Oberkochen, Germany) equipped with a 10× objective and analyzed using dhs Meteor II RGB software (https://physimetrics.com; Dorval, QC, Canada). Three-minute video clips were recorded and analyzed. Cells that moved on the coated recombinant substrates or the endothelial layer that visibly interacted were recorded and grouped into interacting cells (moving between 0–5 μm/s), arresting cells (as a subgroup of the former, staying immobile for ≥1 s), and non-interacting cells. Displacement–time vectors of individual cells were recorded using a 400 × 400 µm grid field (Eppendorf, Hamburg, Germany). For analysis of firm adhesion, MSCs were allowed to arrest on immobilized rhVCAM-1 and analyzed on increasing shear stresses as previously described [2]. Briefly, flow chambers were pre-coated with rhVCAM-1 for 1–2 h, and chemokines were added or not 15 min prior to the experiments at 1 µg/mL. Chambers were flushed once with HBSS/0.05% BSA, and 10^5^ MSCs in HBSS/0.05% BSA were added for 10 min. Shear flow was initiated and increased stepwise at intervals of 2 min. Numbers of attached MSCs were recorded in microscopic fields from photographs taken before, during, and at the end of increasing the shear stress. Photographs of representative fields were recorded at the end of each interval using the CCD camera, and adherent cells were enumerated.

### 2.5. Animal Experiments

siRNA-treated MSCs grown to a confluence of 80% in 6-well plates as described above were trypsinized and labeled with either PKH 26 or 67 fluorescence membrane dye (Sigma, Steinheim, Germany) as described in [14]. Either Rap1A+B siRNA-treated MSCs were labeled with PKH26 and scrambled siRNA controls with PKH67 or vice versa. The viability of labeled MSCs was determined by Trypan blue and found to be ≥95%. Flow-cytometric analysis demonstrated a >95% labeling rate of MSCs with PKH dye in the live cell gates prior to i.v. injection. Single-cell suspensions of fluorescence-labeled cells were pelleted at 400 g for 5 min and resuspended at 1 × 10^6^ /100 μL in 100 U/mL Heparin-Natrium (Ratiopharm, Ulm, Germany) in PBS. The labeled cells were mixed immediately prior to the experiments at 37 °C and injected into the retro-orbital venous plexus of 12- to 16-week-old NOD/SCID mice (Charles River Laboratories, Sulzfeld, Germany) in a protocol approved by the local animal welfare committee. Mice were sacrificed 2 h after transplantation, and single-cell suspensions were prepared from different tissues. MSCs were detected using flow cytometry as previously described [2]. One hundred thousand events were regularly acquired, and the frequency of PKH26+ and PKH67+ cells was determined. Non-transplanted mice were included as controls in every experiment and showed no detectable events in the PKH26- and PKH67-positive gates. Injections and sacrificing of mice were performed under general anesthesia by exposure of mice to a saturated atmosphere of isoflurane in air and under a procedure approved by the local Animal Care Committee. For the visualization of homed MSCs, 5 × 10^6^ PKH-labeled MSCs (controls and siRNA-treated) were co-injected.

### 2.6. Statistical Analysis

A two-sided Student’s *t*-test was performed using Microsoft Office Excel version 2007, assuming normal distribution. In the mouse experiment, the number of detected homed cells in all tissues was set to 100% for the control and the Rap1A+B siRNA groups, and relative contributions within each tissue were analyzed for statistical significance. Values of *p* ≤ 0.05 were considered as significant.

## 3. Results

### 3.1. Expression and Regulation of GTPase Rap1 in MSCs

**Figure 1:**
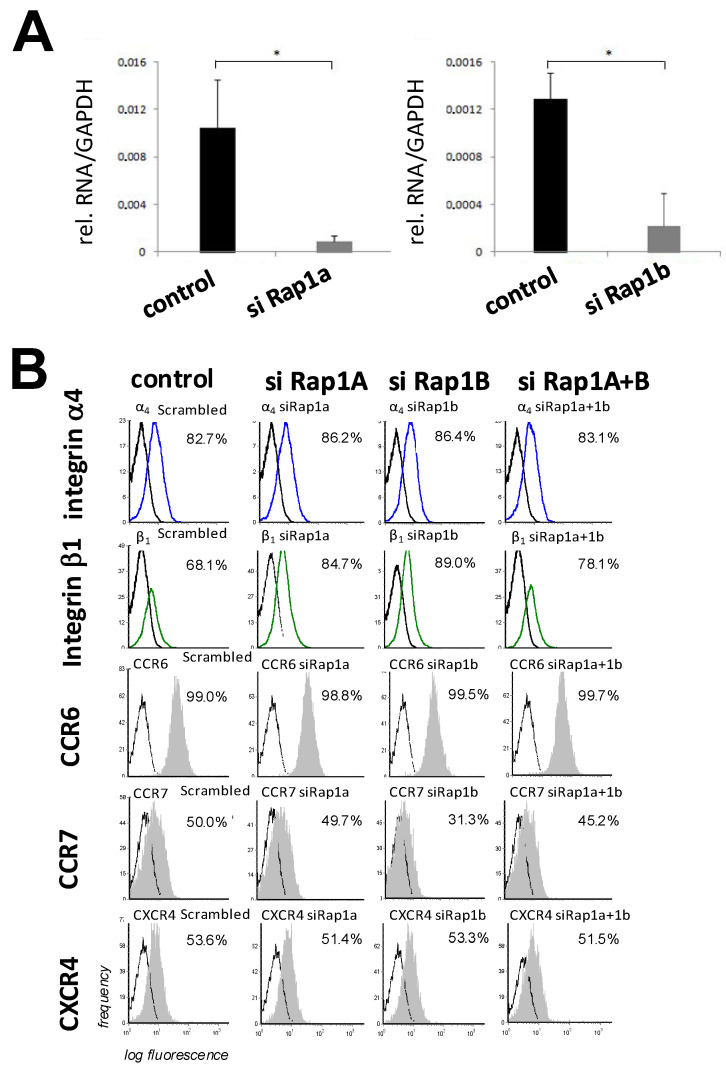
Expression and activation of Rap1 in MSCs. (**A**) Expression of Rap1A and B in MSCs, and downregulation of Rap1A and Rap1B by siRNAs. MSCs were transfected with siRNAs as described in Materials and Methods and analyzed by qRT-PCR 48 h later. (**B**) Analysis of expression of integrins and chemokine receptors in MSCs after siRNA transfection. MSCs were treated with the indicated siRNAs. Forty-eight hours later, cells were detached and analyzed flow-cytometrically for the indicated antigens. Black lines indicate isotype controls and colored lines and grey-shaded curves indicate analyzed receptors. Values and error bars in A and B represent means ± SD of three independent experiments each. *, statistically significant (*p* < 0.05).

We first investigated the expression of Rap1A and B isoforms in MSCs and their modulation by siRNAs. Rap1A and B mRNAs were both detectably expressed in control MSCs (Figure 1A). MSCs treated with siRNA against Rap1A or B displayed > 5-fold decreased levels of Rap1A or B RNA as compared with controls (Figure 1A). We next examined the expression levels of adhesion receptors integrin α4 and integrin β1 and the chemokine receptors CCR6, CCR7, and CXCR4, which are known to be involved in modulating integrin avidity upon stimulation. The expression levels remained constant, irrespective of the modulation of the Rap1 expression (Figure 1B).

### 3.2. Downregulation of Rap1 GTPase Alters Adhesion Behavior of MSCs on HUVECs

**Figure 2:**
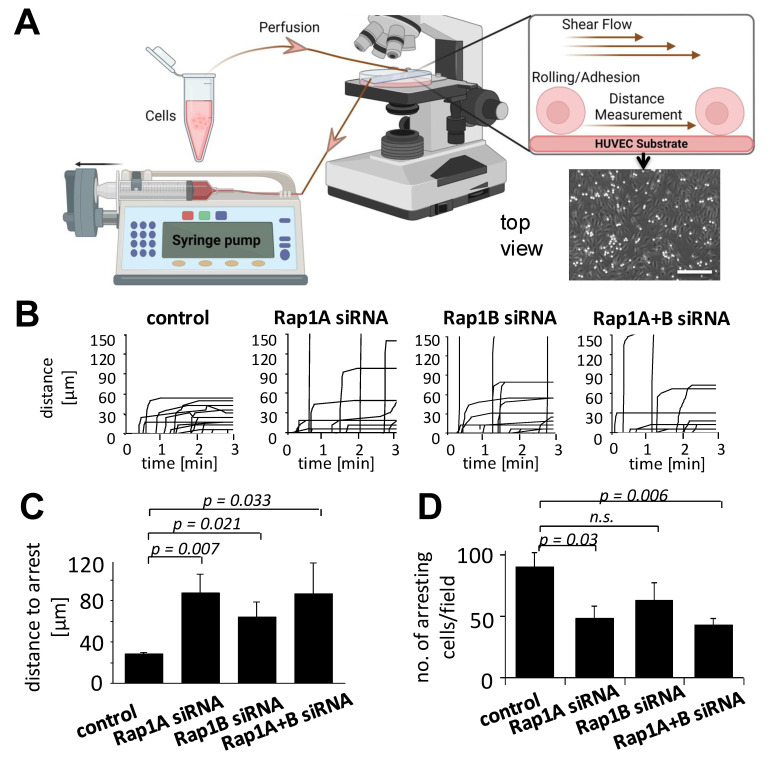
Modulation of Rap1 GTPases affects arrest behavior of MSCs on HUVECs. Ten to the fifth MSCs were treated with siRNAs directed against Rap1A and/or Rap1B or control siRNA for 24 h. MSCs were flushed over HUVEC monolayers pre-stimulated with 10 ng/mL TNF-α overnight and 1 µg/mL CXCL12 for 5 min before the start in parallel-plate flow chambers at a calculated shear stress of 0.35 dyn/cm^2^. (**A**) Schematic layout of the experiment and a representative microscopic field with phase-bright MSCs on the HUVEC layer. Scale bar, 100 µm. (**B**) Time–displacement vectors of individual evaluated cells for each experimental condition. (**C**,**D**) Quantitative evaluation of numbers of arrested cells (**C**) and distances until arrest (**D**) of 9 to 15 individual cells per treatment group. For all experiments, values are means ± SD from 3–5 experiments, with cells from 3 different donors. N.s., not significant. The schema in (**A**) was created using BioRender.

We next investigated the capacity of Rap1-modulated MSCs to interact with endothelial cells under conditions of shear stress. MSCs were flushed over pre-coated, TNF-α pre-stimulated HUVECs in parallel-plate flow chambers, allowing the interaction of MSCs with the endothelial layer and, subsequently, arrest (Figure 2A). Analysis of individual time–displacement vectors of Rap1-modulated MSCs showed that after Rap1 downregulation, part of the MSCs interacted with endothelial cells but did not come to arrest within the microscopic field, whereas all control MSCs arrested within an interaction distance of up to 60 µm (Figure 2B). Control MSCs arrested on HUVECs after an average distance of about 25 µm between the first interaction and final arrest, whereas Rap1A- and/or B-modulated MSCs showed increased means of 80 µm and 60 µm distances (Figure 2C). In parallel, both Rap1A and Rap1A+B-depleted MSCs showed a statistically significant decrease in the number of arrested cells (Figure 2D). Thus, Rap1 depletion interferes with the adhesion behavior of MSCs on HUVEC endothelial cells in vitro by delaying and reducing their arrest in our flow chamber model.

### 3.3. Rap1 Regulates Migration and Firm Arrest of MSCs

**Figure 3:**
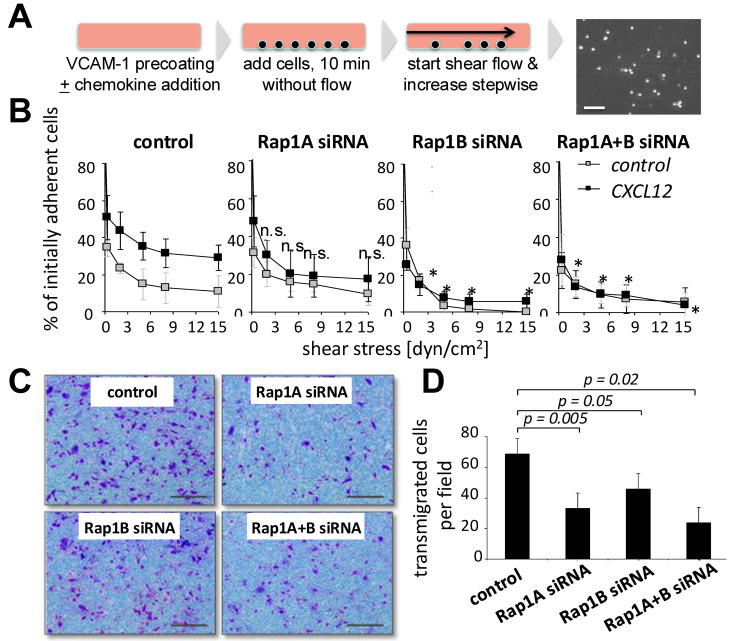
Depletion of Rap1 GTPase affects chemokine-induced firm adhesion and transmigration of MSCs. (**A**) Schematic layout of the experiment was as in Figure 2A, except that cells arrested on pre-coated VCAM-1 ± chemokines and were analyzed after stepwise increasing shear stress. (**B**) Influence of siRNA treatment of MSCs on firm adhesion on immobilized VCAM-1. Forty-eight hours after transfection with the indicated siRNAs, 10^5^ MSCs were allowed to adhere for 3 min on VCAM-1 (2 µg/mL) without (open symbols) or with (closed symbols) co-coating of 1 µg/mL CXCL12. Shear stress was applied by stepwise increases as indicated, and remaining cells were recorded as described in the Methods section. (**C**,**D**) Effect of Rap1A and B downregulation in MSCs on Transwell migration. Forty-eight hours after lipofection with the indicated siRNAs, MSCs were seeded into the upper wells of chemotaxis chambers and allowed to migrate against a gradient of 1% human plasma in DMEM present in the lower wells. (**C**) Typical microscopic views and (**D**) quantitative analysis of bromothymol blue-stained cells on the lower filter sides in microscopic fields. Values are means ± SD; from 5 (**B**) or 3. Scale bar, 100µm (**D**) independent experiments using cells from 3 different donors. * indicates statistically significant difference from control group (*p* < 0.05). N.s., not significant.

To study the ability of MSCs to adhere in immobilized ligands of integrin α4β1, we next allowed Rap1 siRNA-treated MSCs and control MSCs to settle on previously immobilized VCAM-1 in the absence of flow (Figure 3A). When a shear stress of 0.3 dyn/cm^2^ was applied, approximately 30% of MSCs remained adherent on VCAM-1, and after stepwise increases in shear stress, approximately half of the MSCs de-adhered, and about 15% of initially adherent MSCs remained adherent at the highest shear stress of 15 dyn/cm^2^ (Figure 3B). In parallel experiments, CXCL12 was co-immobilized with VCAM-1; 60% of control MSCs resisted the lowest shear, and 45% remained adherent after high shear stress (at 15 dyn/cm^2^). MSCs treated with Rap1A siRNA were found to adhere at comparable levels as controls in the absence of CXCL12 and to a lesser degree in the presence of CXC12 (Figure 3A; not statistically significant). In contrast, after the downregulation of either Rap1B alone or Rap1A+B in combination, CXCL12-induced adhesion was strongly and significantly reduced (Figure 3B). Non-CXCL12-stimulated adhesion was also reduced in Rap1B or Rap1A+B siRNA-treated MSCs compared with control MSCs, but this did not reach statistical significance. We next assessed the transmigration of MSCs using a Transwell assay in a gradient of CXCL12. MSCs transfected with siRNA against Rap1A and/or B genes all showed significantly diminished migration in Transwell assays (Figure 3C,D). Together, modulation of both Rap1A and Rap1B affected transmigration, whereas CXCL12-induced firm adhesion depended mostly on Rap1B in MSCs.

### 3.4. Downregulation of Rap1A+B Affects Tissue Distribution of Fluorescence-Marked MSCs in Immune-Deficient Mice

**Figure 4:**
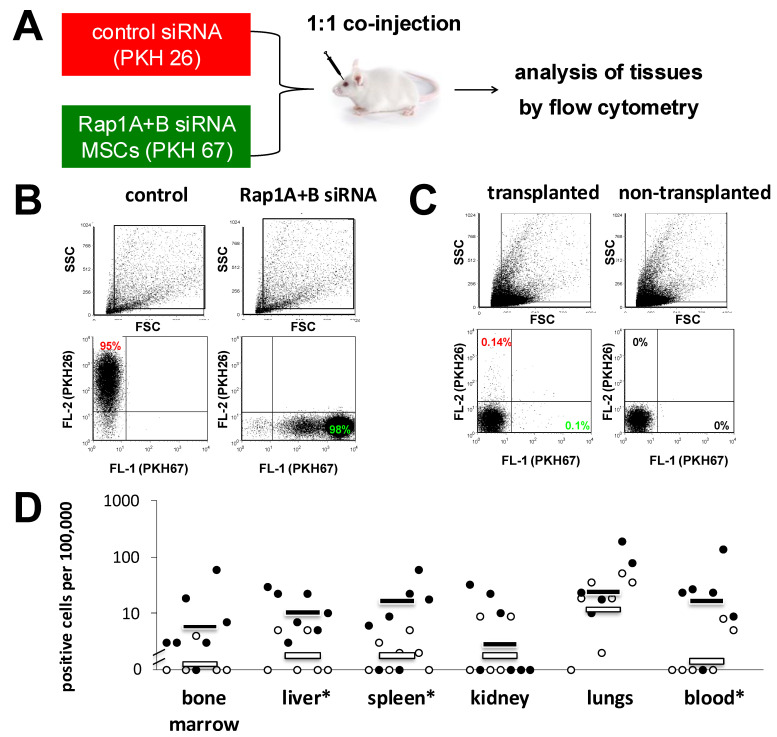
Tissue distribution of Rap1-modulated MSCs in mice. (**A**) Experimental layout. MSCs were lipofected with scrambled sequence (control) or Rap1A+B siRNAs. Forty-eight hours later, control or Rap1A+B siRNA-treated MSCs were labeled with PKH 26 or 67 fluorescence dyes as described in Materials and Methods. Ten to the sixth of control and of knockdown MSCs in 100 µL each were mixed immediately before injection and co-injected i.v. into NOD/SCID mice. Two hours later, mice were euthanized, and cell suspensions were prepared from blood and the indicated tissues and analyzed by flow cytometry. (**B**,**C**) Representative flow-cytometric results of labeled MSCs prior to injection (**B**) and in the lung of a transplanted and of a non-transplanted mouse (**C**). (**D**) Quantitative evaluation of detected cells per tissue. Values are means ± SD in % of detected MSCs per 100,000 cells in the indicated mouse tissues from a total of seven mice analyzed in six independent experiments using MSCs from 3 different donors. White dots, controls; black dots, Rap1A+B siRNA treated. Asterisks indicate statistically significant differences (*p* < 0.05) between the treated and control group.

To investigate whether the downregulation of Rap1 genes affects the circulation of transplanted MSCs in a short-term murine homing model, we co-injected 1:1 mixtures (1 × 10^6^ cells each) of Rap1A+B-depleted and control MSCs that were pre-labeled with two fluorescent vital dyes, PKH26 and PKH67, into immunodeficient NOD/SCID mice. Figure 4A shows the experimental layout. PKH^+^ events in both the green (PKH67) and red (PKH26) fluorescence channels were detectable in the MSCs before injection (Figure 4B). Figure 4C shows the detection of positive events in the MSC cell gate in lung cell suspensions of MSCs from transplanted mice. No signals were obtained in mice that received no transplant, demonstrating that the fluorescence was solely derived from injected MSCs (Figure 4C). We found that the highest frequencies of MSCs were detected in the lungs (Figure 4D). Increased frequencies of Rap1A+B siRNA-treated MSCs compared to control MSCs were detected in the blood, bone marrow, liver, and spleen (Figure 4D). This reached statistical significance for spleen, liver, and blood (Figure 4D). Thus, the siRNA-mediated modulation of Rap1A+B in MSCs increased the number of MSCs detected in liver and spleen compared to controls.

## 4. Discussion

MSCs are administered via the intravenous route in many clinical studies, yet knowledge regarding the mechanisms that MSCs employ to regulate their interaction with the vessel wall and tissue distribution is still incomplete. We here provide evidence for a role of the Rap1 GTPase in the adhesion, migration, and short-term bioavailability of MSCs in mice.

So far, only limited data exist on the expression and potential role of Rap1 in MSCs. Two previous studies have suggested a role for Rap1 in MSC biology. Treatment of MSCs with High Mobility Group Box (HMGB)-1 was observed to induce chemokine synthesis and activation of Rap1 in MSCs, suggesting that Rap1 plays a role in MSC migration [20]. In human umbilical cord blood-derived MSCs, a role of a prostaglandin E2 and the Rap1 guanine nucleotide exchange factor EPAC1 was suggested in the proliferation of MSCs [18] and their migration [25]. We confirm that Rap1 is expressed in human MSCs in both A and B isoforms. The functional responses obtained after the siRNA treatment of MSCs indicate a more prominent role of Rap1B in shear stress-dependent adhesion and a role of both isoforms in the transmigration assays.

We and others have previously demonstrated that integrin receptors that target endothelial ligands are a major target of Rap1 signaling in hematopoietic cells and endothelial cells [26,27,28,29]. MSCs, after trypsinization and during injection in the bloodstream, show a spheroid form, acquire cortical actin similar to hematopoietic cells, and can activate integrin receptors to interact with endothelial cells [22,30,31]. Here, we asked which degree Rap1 may regulate MSC migration under shear stress and after intravenous injection in mice. Since it is known that the arrest and subsequent induction of firm adhesion in hematopoietic cells under shear flow depend on the activation of integrins and chemokine receptors [32], we employed a parallel-plate flow chamber model, which allows the study of the integrin-mediated interaction of leukocytes with endothelial ligands or endothelial cells [2,8,28]. We have previously established for our model that the application of either anti-VLA4 or anti-VCAM antibodies abolishes the adhesion of MSCs on HUVECs [2]. Our data show that firm adhesion of MSCs is induced by CXCL12 on recombinant VCAM-1 and that it is reduced after the downregulation of Rap1B. Thus, Rap1B is a major regulator of chemokine-induced integrin activation in MSCs.

In our short-term homing model, the downmodulation of both isoforms of Rap1 resulted in sufficient preservation of integrin activity to allow the accumulation of MSCs in several tissues. The endothelial interaction and extravasation of MSCs in lung and other tissues has been confirmed in our mouse model by immunohistochemistry previously [2,24]. Moreover, we found similar frequencies of MSCs accumulating in the lungs after Rap1 downregulation and in controls, yet higher proportions in blood and several tissues. These data suggest that the circulation behavior of transplanted MSCs is an active process and is not regulated alone by cell size and passive entrapment. Moreover, Rap1 modulation may positively influence the migration of MSCs into tissues in mice.

Masterson et al. have described, using intravital microscopy, that MSCs arrest at pulmonary micro-vessel bifurcations due to size obstruction and release microparticles when captured in the precapillary beds [31]. Similarly, we have previously reported that MSCs are fragmented at a quantitative level after i.v. injection in mice during lung passage [24]. Also in a murine model, Fischer et al. found that the application of function-blocking anti-integrin α4 antibody increased pulmonary passage and positively affected the distribution of MSCs in two separate injections [33]. These data support the observation of improved migration of MSCs to tissues after Rap1 downmodulation.

Our flow chamber model also indicated that control MSCs were not able to de-adhere once they interacted with endothelial cells but were, at least in part, able to de-adhere after the downregulation of Rap1. De-adhesion of arrested cells has been found to be a part of the physiological homing process into the murine bone marrow by hematopoietic stem cells [34]. Studies using intravital microscopy are, therefore, needed to address the question of whether coordinated extravasation of MSCs may occur at increased frequencies after the modulation of Rap1.

## 5. Conclusions

Our data show that the siRNA-mediated downregulation of Rap1 resulted in a decreased, but not abolished, interaction with vascular ligand VCAM-1 or endothelial cells in MSCs. When applying Rap1A+B-depleted MSCs in our in vivo short-term homing model, we observed an increase in the accumulation of MSCs in peripheral tissues. These data argue for a dual role of Rap1 in the circulatory behavior of transplanted MSCs and suggest that the modulation of Rap1 may be beneficial when transplanting MSCs for therapy.

## Data Availability

Data are contained within the article.

## References

[1] Barmada A., Sharan J., Band N., Rumschlag T., Yaqub A., Liebman E., Prodromos C. (2023). Review of the Published Literature Confirms the Safety of Intravenous Infusion of Mesenchymal Stem Cells. Curr. Stem Cell Res. Ther..

[2] Rüster B., Göttig S., Ludwig R.J., Bistrian R., Müller S., Seifried E., Gille J., Henschler R. (2006). Mesenchymal stem cells display coordinated rolling and adhesion behavior on endothelial cells. Blood.

[3] Karp J.M., Teo G.S.L. (2008). Mesenchymal Stem Cell Homing: The Devil Is in the Details. Cell Stem Cell.

[4] Teo G.S., Ankrum J.A., Martinelli R., Boetto S.E., Simms K., Sciuto T.E., Dvorak A.M., Karp J.M., Carman C.V. (2012). Mesenchymal stem cells transmigrate between and directly through tumor necrosis factor-α-activated endothelial cells via both leukocyte-like and novel mechanisms. Stem Cells.

[5] Nitzsche F., Müller C., Lukomska B., Jolkkonen J., Deten A., Boltze J. (2017). Concise Review: MSC Adhesion Cascade-Insights into Homing and Transendothelial Migration. Stem Cells.

[6] Gao Q., Xia Y., Liu L., Huang L., Liu Y., Zhang X., Xu K., Wei J., Hu Y., Mu Y. (2016). Galectin-3 Enhances Migration of Minature Pig Bone Marrow Mesenchymal Stem Cells Through Inhibition of RhoA-GTP Activity. Sci. Rep..

[7] Suila H., Hirvonen T., Kotovuori A., Ritamo I., Kerkelä E., Anderson H., Natunen S., Tuimala J., Laitinen S., Nystedt J. (2014). Human umbilical cord blood-derived mesenchymal stromal cells display a novel interaction between P-selectin and galectin-1. Scand J. Immunol..

[8] Sackstein R. (2012). Glycoengineering of HCELL, the human bone marrow homing receptor: Sweetly programming cell migration. Ann. Biomed. Eng..

[9] Wu Y., Zhao R.C. (2012). The role of chemokines in mesenchymal stem cell homing to myocardium. Stem Cell Rev..

[10] Ciuculescu F., Giesen M., Deak E., Lang V., Seifried E., Henschler R. (2011). Variability in chemokine-induced adhesion of human mesenchymal stromal cells. Cytotherapy.

[11] Semon J.A., Nagy L.H., Llamas C.B., Tucker H.A., Lee R.H., Prockop D.J. (2010). Integrin expression and integrin-mediated adhesion in vitro of human multipotent stromal cells (MSCs) to endothelial cells from various blood vessels. Cell Tissue Res..

[12] Gronthos S., Simmons P.J., Graves S.E., Robey P.G. (2001). Integrin-mediated interactions between human bone marrow stromal precursor cells and the extracellular matrix. Bone.

[13] Zwolanek D., Flicker M., Kirstätter E., Zaucke F., van Osch G.J., Erben R.G. (2015). β1 Integrins Mediate Attachment of Mesenchymal Stem Cells to Cartilage Lesions. Biores. Open Access.

[14] Wang X., Tang P., Guo F., Zhang M., Yan Y., Huang M., Chen Y., Zhang L., Zhang L. (2019). mDia1 and Cdc42 Regulate Activin B-Induced Migration of Bone Marrow-Derived Mesenchymal Stromal Cells. Stem Cells.

[15] Ge J., Burnier L., Adamopoulou M., Kwa M.Q., Schaks M., Rottner K., Brakebusch C. (2018). RhoA, Rac1, and Cdc42 differentially regulate αSMA and collagen I expression in mesenchymal stem cells. J. Biol. Chem..

[16] Melzer M., Niebert S., Heimann M., Ullm F., Pompe T., Scheiner-Bobis G., Burk J. (2024). Differential Smad2/3 linker phosphorylation is a crosstalk mechanism of Rho/ROCK and canonical TGF-β3 signaling in tenogenic differentiation. Sci. Rep..

[17] Li C., Zhen G., Chai Y., Xie L., Crane J.L., Farber E., Farber C.R., Luo X., Gao P., Cao X. (2016). RhoA determines lineage fate of mesenchymal stem cells by modulating CTGF-VEGF complex in extracellular matrix. Nat. Commun..

[18] Jang M.W., Yun S.P., Park J.H., Ryu J.M., Lee J.H., Han H.J. (2012). Cooperation of Epac1/Rap1/Akt and PKA in prostaglandin E(2) -induced proliferation of human umbilical cord blood derived mesenchymal stem cells: Involvement of c-Myc and VEGF expression. J. Cell Physiol..

[19] Zhang Y., Chiu S., Liang X., Gao F., Zhang Z., Liao S., Liang Y., Chai Y.H., Low D.J., Tse H.F. (2015). Rap1-mediated nuclear factor-kappaB (NF-κB) activity regulates the paracrine capacity of mesenchymal stem cells in heart repair following infarction. Cell Death Discov..

[20] Lin F., Xue D., Xie T., Pan Z. (2016). HMGB1 promotes cellular chemokine synthesis and potentiates mesenchymal stromal cell migration via Rap1 activation. Mol. Med. Rep..

[21] Leibacher J., Henschler R. (2016). Biodistribution, migration and homing of systemically applied mesenchymal stem/stromal cells. Stem Cell Res. Ther..

[22] Jaganathan B.G., Ruester B., Dressel L., Stein S., Grez M., Seifried E., Henschler R. (2007). Rho inhibition induces migration of mesenchymal stromal cells. Stem Cells.

[23] Duchniewicz M., Zemojtel T., Kolanczyk M., Grossmann S., Scheele J.S., Zwartkruis F.J. (2006). Rap1A-deficient T and B cells show impaired integrin-mediated cell adhesion. Mol. Cell. Biol..

[24] Leibacher J., Dauber K., Ehser S., Brixner V., Kollar K., Vogel A., Spohn G., Schäfer R., Seifried E., Henschler R. (2017). Human mesenchymal stromal cells undergo apoptosis and fragmentation after intravenous application in immune-competent mice. Cytotherapy.

[25] Yu J.L., Deng R., Chung S.K., Chan G.C. (2016). Epac Activation Regulates Human Mesenchymal Stem Cells Migration and Adhesion. Stem Cells.

[26] Bos J.L., de Rooij J., Reedquist K.A. (2001). Rap1 signaling: Adhering to new models. The small GTPase Rap1 is a potent activation signal for beta1, beta2, and beta3 integrins and enhances cellular adhesion in both immune and nonimmune cells. Nat. Rev. Mol. Cell Biol..

[27] Bivona T.G., Wiener H.H., Ahearn I.M., Silletti J., Chiu V.K., Philips M.R. (2004). Rap1 up-regulation and activation on plasma membrane regulates T cell adhesion. J. Cell Biol..

[28] Ebisuno Y., Katagiri K., Katakai T., Ueda Y., Nemoto T., Inada H., Nabekura J., Okada T., Kannagi R., Tanaka T. (2010). Rap1 controls lymphocyte adhesion cascade and interstitial migration within lymph nodes in RAPL-dependent and -independent manners. Blood.

[29] Carmona G., Göttig S., Orlandi A., Scheele J., Bäuerle T., Jugold M., Kiessling F., Henschler R., Zeiher A.M., Dimmeler S. (2009). Role of the small GTPase Rap1 for integrin activity regulation in endothelial cells and angiogenesis. Blood.

[30] Teo G.S., Yang Z., Carman C.V., Karp J.M., Lin C.P. (2015). Intravital imaging of mesenchymal stem cell trafficking and association with platelets and neutrophils. Stem Cells.

[31] Masterson C.H., Tabuchi A., Hogan G., Fitzpatrick G., Kerrigan S.W., Jerkic M., Kuebler W.M., Laffey J.G., Curley G.F. (2021). Intra-vital imaging of mesenchymal stromal cell kinetics in the pulmonary vasculature during infection. Sci. Rep..

[32] Alon R., Rose D.M., Ginsberg M.H. (2007). Integrin modulation and signaling in leukocyte adhesion and migration. Immunol. Rev..

[33] Fischer U.M., Harting M.T., Jimenez F., Monzon-Posadas W.O., Xue H., Savitz S.I., Laine G.A., Cox C.S. (2009). Pulmonary passage is a major obstacle for intravenous stem cell delivery: The pulmonary first-pass effect. Stem Cells Dev..

[34] Mazo I.B., Gutierrez-Ramos J.C., Frenette P.S., Hynes R.O., Wagner D.D., von Andrian U.H. (1998). Hematopoietic progenitor cell rolling in bone marrow microvessels: Parallel contributions by endothelial selectins and vascular cell adhesion molecule 1. J. Exp. Med..

